# Fengshi Gutong Capsule Attenuates Osteoarthritis by Inhibiting MAPK, NF-κB, AP-1, and Akt Pathways

**DOI:** 10.3389/fphar.2018.00910

**Published:** 2018-08-17

**Authors:** Yao-Xin Gao, Hao-Heng Yu, Chuan He, Ming Li, Dan-Dan Guo, Jun-Jiang Lian, Hai-Jie Yang, Mian Wang, Lei Wang, Zhi-Wei Feng, Bin-Feng Cheng

**Affiliations:** ^1^School of Life Sciences and Technology, Xinxiang Medical University, Xinxiang, China; ^2^Henan Key Laboratory of Medical Tissue Regeneration, Xinxiang Medical University, Xinxiang, China; ^3^School of Basic Medical Sciences, Xinxiang Medical University, Xinxiang, China; ^4^The First Affiliated Hospital of Xinxiang Medical University, Xinxiang, China

**Keywords:** Fengshi Gutong capsule, osteoarthritis, rat OA model, synovial cells, pathways

## Abstract

**Background and purpose:** Fengshi Gutong capsule (FSGTC), a traditional herbal formula, has been used clinically in China for the treatment of arthritis. However, the mechanism underlying the therapeutic effects of FSGTC on osteoarthritis (OA) has not been elucidated. The present study investigated the function and mechanisms of FSGTC in rat OA model and interleukin (IL)-1β-stimulated synovial cells.

**Materials and methods:** Rat OA model was established by intra-articular injection containing 4% papain. IL-1β-induced SW982 cells were used as an OA cell model. Safranin-O-Fast green (S-O) and hematoxylin-eosin (HE) stainings were used to observe the changes in cartilage morphology. Enzyme-linked immunosorbent assay (ELISA) and real-time quantitative PCR (qPCR) detected the expression of inflammatory cytokines. In addition, molecular mechanisms were analyzed by Western blot in the OA cell model.

**Results:** FSGTC treatment significantly relieved the degeneration of cartilage and reduced the contents of tumor necrosis factor-α (TNF-α) and IL-6 in the serum in papain-induced OA rats. FSGTC also reduced the protein and mRNA levels of IL-6 and IL-8 in IL-1β-stimulated SW982 cells. Moreover, it inhibited the phosphorylation levels of ERK (extracellular signal-related kinase), JNK (c-Jun N-terminal kinase), p38, Akt (protein kinase B), and c-Jun. It also decreased the extent of IκBα degradation and p65 protein translocation into the nucleus.

**Conclusion:** The current data confirmed the protective effects of FSGTC in the rat and OA cell models. The results suggested that FSGTC reduced the production of inflammatory mediators via restraining the activation of mitogen-activated protein kinases (MAPK), nuclear factor kappa B (NF-κB), activator protein-1 (AP-1), and Akt.

## Introduction

Osteoarthritis (OA) is the most common form of arthritis, resulting in mental stress, disability, physical limitations, and socioeconomic burden (French et al., 2016). The presence of inflammatory factors, such as interleukin (IL)-1β and tumor necrosis factor-α (TNF-α), causes significant symptoms of arthritic pain and leads to the putative disease progression that has affected approximately 1% population worldwide (Sellam and Berenbaum, 2010; Lee et al., 2013); the morbidity of OA in women is about threefold higher than that in men (Kameda, 2012). Any single drug that can control and treat OA has not yet been discovered. The primary drugs on the market for OA treatment are disease-modifying anti-rheumatic drugs, biological agents, non-steroidal anti-inflammatory drugs, and glucocorticoids. However, these anti-inflammatory drugs exert several adverse effects and do not completely alleviate the chronic inflammation. Previous studies have focused on the discovery of new and effective anti-inflammatory substances with low adverse effects (Tabas and Glass, 2013).

Traditional Chinese medicine (TCM) is widely utilized in China for several years, and it offers a holistic approach to treat various diseases. Herbal products are the most commonly used forms in TCM (Manheimer et al., 2009). The effect of herbal formulas on OA has been demonstrated as an integrated result of various mechanisms of action, such as inflammatory control and immunity adjustment (Xu et al., 2017). Herbal formulae synthesize a variety of medicinal properties in the treatment of OA such that only a few side effects are exhibited, and hence, they can be used for prolonged periods (Wang et al., 2017). Thus, the herbal formulae are speculated to have a great potential as effective therapeutic agents for OA (Li et al., 2017).

Fengshi Gutong capsule (FSGTC) is a traditional herbal formula that has been widely sold to treat OA in many hospitals and drugstores. It is approved by the China Food and Drug Administration (No. Z34020025) and is composed of seven herbs including Aconiti Radix Cocta (boiled root of *Aconitum carmichaelii* Debx.), Aconiti Kusnezoffii Radix Cocta (boiled root of *Aconitum kusnezoffii* Reichb.), Carthami Flos (flower of *Carthamus tinctorius* L.), Glycyrrhizae Radix Et Rhizoma (root and rhizome of *Glycyrrhiza uralensis* Fisch.), Chaenomelis Fructus [fructus of *Chaenomeles speciosa* (Sweet) Nakai], Mume Fructus [fructus of *Prunus mume* (Sieb.) Sieb. et Zucc.], and Ephedrae Herba (rhizome of *Ephedra sinica* Stapf). There have been some reports about the anti-inflammatory properties of the major components of FSGTC, such as liquiritin and kaempferol could inhibit the inflammatory response in rheumatoid arthritis model (Lian et al., 2016; Gao et al., 2017). Clinical observations demonstrated that FSGTC reduces the pain of the lesion area, improves the function of the joint, and relieves the swelling of the joint or numbness of the limbs (Shen, 2002; Chen et al., 2009; Wang and Wang, 2009). The results of animal model experiments indicated that FSGTC could significantly reduce the rate of foot swelling in the rat OA model (Tian and Li, 2016). However, the molecular mechanisms underlying FSGTC are yet to be elucidated.

In this study, we studied the function and mechanism of FSGTC in rat OA model and IL-1β-stimulated SW982 synovial cells. Furthermore, the potential anti-inflammatory mechanisms underlying FSGTC were also investigated.

## Materials and Methods

### Chemicals and Reagents

Interleukin-1β was obtained from PeproTech (Rocky Hill, NJ, United States). Roswell Park Memorial Institute (RPMI) 1640 medium was purchased from Thermo Fisher Scientific Inc. (Logan, UT, United States). Antibodies against p-ERK (extracellular signal-related kinase), p-p38 MAPK (mitogen-activated protein kinase), p-JNK (c-Jun N-terminal kinase), NF-κB (nuclear factor kappa B) (p65), p-p65, I kappa B alpha (IκBα), p-c-Jun, p-Akt (protein kinase B), lamin B, and actin were purchased from Cell Signaling Technology Inc. (Beverly, MD, United States). TRIzol reagent was obtained from Invitrogen (Carlsbad, CA, United States). QuantiTect Reverse Transcription kit was purchased from Qiagen (Valencia, CA, United States). SYBR Green Master Mix was purchased from Bio-Rad Laboratories (Hercules, CA, United States). 0.25% trypsin ethylenediaminetetraacetic acid (trypsin-EDTA) was purchased from Gibco (Life Technologies Co., Carlsbad, CA, United States). Fetal bovine serum (FBS) and cell lysates were obtained from Sigma-Aldrich (St. Louis, MO, United States). The secondary antibodies were procured from Santa Cruz Biotechnology (Santa Cruz, CA, United States). All other reagents were obtained from Sigma Chemical Co. unless otherwise stated.

### Preparation of FSGTC

Commercial FSGTC was purchased from Anhui Jing Fang Pharmaceutical Ltd. (Anhui, China, batch No. 151003). For quality control, five major components of FSGTC were analyzed and determined by high performance liquid chromatography (HPLC), which included ursolic acid (1.263 mg/g), kaempferol (0.198 mg/g), ephedrine (0.368 mg/g), hydroxysafflor yellow A (0.155 mg/g), and glycyrrhizic acid (6.654 mg/g).

Fengshi Gutong capsule powder was suspended and diluted with physiological saline for administration to rats. For *in vitro* experiments, the FSGTC powder was extracted with 50% ethanol and solubilized in <0.1% dimethyl sulfoxide (DMSO).

### Papain-Induced Rat OA Model and Drug Administration

Wistar rats (male, 180–220 g) were purchased from the Experimental Animal Center of the National Institute for the Control of Pharmaceutical and Biological Products (Beijing, China). All animals were housed in a temperature-controlled room (22 ± 2°C) and allowed free access to standard pelleted forage and tap water. All rats were fed for 7 days for acclimatization before experiments. All animal experiments were performed in accordance with the Animal Ethics Committee of Xinxiang Medical University.

After adaptive feeding, the rats were randomly assigned to four groups: sham, model, FSGTC low-dose (F-L; 200 mg/kg), and FSGTC high-dose (F-H; 400 mg/kg). FSGTC was administered orally to rats daily for 1 week before papain injection. For papain-induced rat OA model, the right knee joint of the rat was injected with 0.2 mL of 4% papain every 3 days, three times, while the sham group received an equivalent volume of sterile saline solution.

### Histological Analysis

Knee joint samples were fixed in 4% paraformaldehyde for 24 h at 4°C and decalcified in 10% EDTA solution at 4°C for 2 weeks. Then, the samples were dehydrated by graded alcohol, clarified, and embedded in paraffin blocks. Frontal serial sections (4-μm thick) across the entire joint were obtained, and 10 slides/joint at every 40 μm were selected and stained with Safranin-O-Fast green (S-O) and hematoxylin-eosin (HE) to detect the cartilage destruction. The images were captured digitally by a microscope.

### Determination of Cytokines Levels in Serum

Blood samples were collected after the final administration, allowed to coagulate at room temperature, and centrifuged at 3500 rpm for 15 min. Consequently, the serum was separated and stored at -80°C. The concentrations of inflammatory cytokines (TNF-α and IL-6) in the serum were estimated using enzyme-linked immunosorbent assay (ELISA) kits (Pierce Endogen, Rockford, IL, United States) according to the manufacturer’s instructions.

### Cell Cultures

The human synovial sarcoma cell line SW982 was obtained from the American Type Culture Collection (Rockville, MD, United States) and cultured in RPMI 1640 medium supplemented with 10% FBS and 1% penicillin-streptomycin at 37°C in 5% CO_2_ for several days. The medium was changed every 2 days.

### ELISA

The SW982 cells were treated with various doses (200 and 400 mg/mL) of FSGTC for 30 min, and then, 10 ng/mL of IL-1β was added to each well. After 12 h, the supernatants were collected. The concentrations of the inflammatory cytokines (IL-6 and IL-8) in the medium were measured using ELISA kits, according to the manufacturer’s instructions.

### Real-Time PCR

The total RNA was collected using TRIzol reagent according to the manufacturer’s instructions, and cDNA was synthesized using the High-Capacity cDNA Reverse Transcription Kit. RT-PCR was performed on ABI Prism 7500 Sequence detection system (Applied Biosystems, Foster City, CA, United States) using the KAPA SYBR qPCR kit according to the manufacturer’s instructions. The primers for *IL-6*, *IL-8*, and *GAPDH* for RT-PCR are listed in **Table 1**.

**Table 1:** Primers for real-time PCR.

Gene	Primer	Sequence (5′–3′)	Product size (bp)
IL-6	Forward	TACATCCTCGACGGCATCTCA	165
	Reverse	CACCAGGCAAGTCTCCTCATTG	
IL-8	Forward	TCTTGGCAGCCTTCCTGATT	152
	Reverse	TGGTCCACTCTCAATCACTCTCAGT	
GAPDH	Forward	TGCCCTCAACGACCACTTTG	105
	Reverse	TACTCCTTGGAGGCCATGTG	

### Western Blot

The cells were lysed in lysis buffer, followed by centrifugation at 12,000 rpm for 10 min to collect the supernatants. After determining the protein concentrations by the Bio-Rad protein assay, the samples were subjected to sodium-dodecyl sulfate polyacrylamide gel electrophoresis (SDS-PAGE) and transferred to polyvinylidene difluoride filter (PVDF) membranes (Millipore, Bedford, MA, United States). The membranes were blocked with 5% dried skim milk in Tris-buffered saline/Tween 20 (TBST) for 1 h at room temperature, followed by probing with primary antibodies against p-ERK, p-p38, p-JNK, NF-κB (p65), IκBα, p-p65, p-c-Jun, p-Akt, lamin B, and actin overnight at 4°C. Subsequently, the membranes were washed three times with TBST and incubated with the secondary antibody for 40 min at room temperature. The density of the immunoreactive bands was measured using a computer-assisted image analysis system (Adobe Systems, San Jose, CA, United States). Nuclear and cytoplasmic extracts were prepared using the nuclear protein extraction kit (BestBio, China); the changes in the expression levels of nuclear p65 and IκBα in SW982 cells treated with IL-1β were observed.

### Statistical Analysis

All data were expressed as mean ± SD. Data were compared using the one-way ANOVA test. *p* < 0.05 Indicated statistically significant difference. Each experiment consisted of at least three replicates per condition.

## Results

### Effects of FSGTC on Cartilage Damage in a Rat OA Model

**FIGURE 1:**
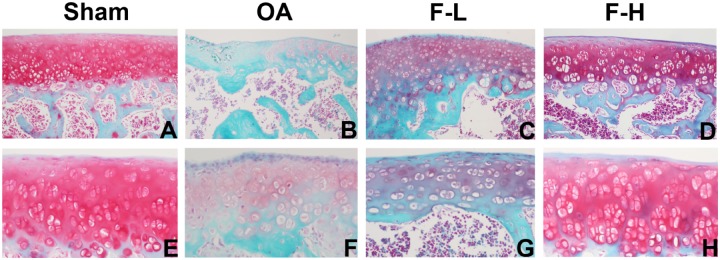
Effects of FSGTC on the destruction of cartilage in OA rats, assayed by S-O staining. Rats were treated without **(B,F)** or with 200 mg/kg **(C,G)** and 400 mg/kg **(D,H)** FSGTC before papain injection; sterile saline solution injection was used for the sham group **(A,E)** (original magnification ×100 or ×200).

**FIGURE 2:**
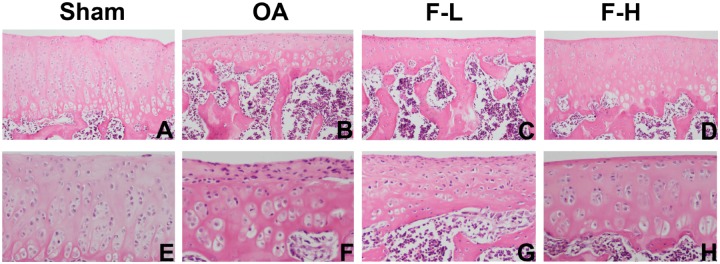
Effects of FSGTC on the destruction of cartilage in OA rats assayed by HE staining. Rats were treated without **(B,F)** or with 200 mg/kg **(C,G)** and 400 mg/kg **(D,H)** of FSGTC before papain injection; sterile saline solution injection was used for the sham group **(A,E)** (original magnification ×100 or ×200).

In order to determine whether FSGTC exerts a protective effect against the occurrence and development of OA *in vivo*, the rat OA models were established. The histological analysis of OA was examined by S-O and HE staining. As shown in **Figure 1**, S-O staining for proteoglycans showed a significant reduction in the cartilage matrix in the model group as compared to the sham group, indicating the matrix degradation of OA. However, the matrix degradation in the FSGTC low-dose and FSGTC high-dose groups was less than that in the model group. Concomitantly, HE staining showed that the articular cartilage in the sham group had a regular morphological structure, while the model group exhibited a reduction in chondrocytes and articular cartilage thickness with an irregular morphological structure (**Figure 2**). On the other hand, the FSGTC low-dose and FSGTC high-dose groups led to an obvious increase in the amelioration of cartilage damage and articular cartilage thickness.

### Effects of FSGTC on the Levels of Cytokines in the Serum of OA Rats

**FIGURE 3:**
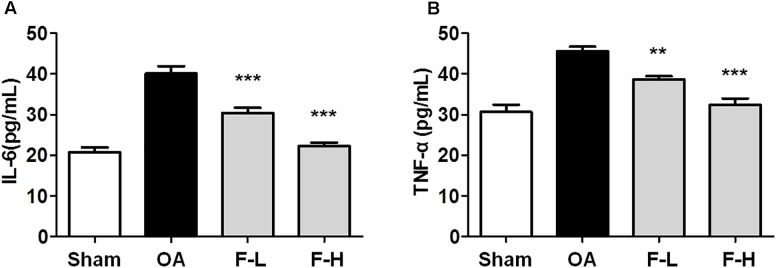
Effects of FSGTC on serum levels of TNF-α **(A)** and IL-6 **(B)**. Rats were orally treated with various doses (200 and 400 mg/kg) of FSGTC. Subsequently, serum was sampled, separated, and concentration determined using ELISA kits. Data are represented as mean ± SD. Statistical analyses were performed by one-way ANOVA test, followed by Dunnett’s *post hoc* test. *n* = 8, *x* ±*s*. ^∗^*p* < 0.05, ^∗∗^*p* < 0.01, ^∗∗∗^*p* < 0.001 vs. IL-1β without FSGTC.

The serum levels of IL-6 and TNF-α were determined by ELISA, according to the manufacturer’s instructions. After the addition of FSGTC, the release of IL-6 and TNF-α was decreased sharply in the serum (**Figures 3A,B**). These results demonstrated that FSGTC might promote the anti-inflammatory function in rat OA model.

### Effects of FSGTC on the Production of Inflammatory Cytokines IL-6 and IL-8 in IL-1β-Induced SW982 Cells

**FIGURE 4:**
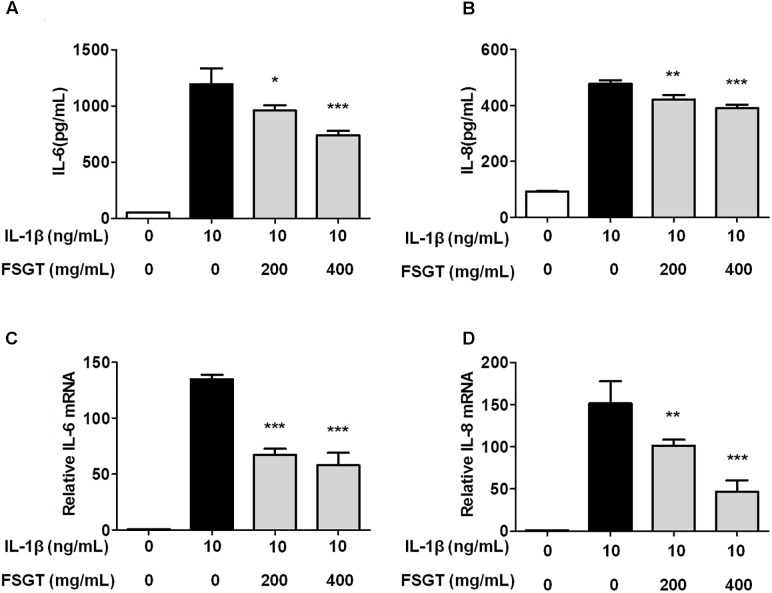
Effects of FSGTC on IL-1β-induced protein levels of IL-6 **(A)** and IL-8 **(B)** as well as mRNA expression of *IL-6* **(C)** and *IL-8* **(D)** in SW982 cells. The cells were treated with various doses (200 and 400 mg/mL) of FSGTC. The supernatants were collected and analyzed for IL-6 and IL-8 by ELISA kits. The total RNA was collected and prepared for qPCR of *IL-6* **(C)** and *IL-8* **(D)** from SW982 cells. *GAPDH* was used as an internal control to normalize the data. Data are presented as the mean ± SD. Statistical analyses were performed by one-way ANOVA test, followed by Dunnett’s *post hoc* test. *n* = 3, *x* ±*s*. ^∗^*p* < 0.05; ^∗∗^*p* < 0.01, ^∗∗∗^*p* < 0.001 vs. IL-1β without FSGTC.

To examine the protective effect of FSGTC on the expression of inflammatory cytokines, SW982 cells were treated with different doses (200 and 400 mg/mL) of FSGTC for 30 min before stimulation with IL-1β (10 ng/mL). The supernatants were collected after 12 h. The levels of IL-6 and IL-8 were detected by ELISA. After IL-1β stimulation, SW982 cells showed an apparent inflammatory reaction. As a result of FSGTC, the secretion of IL-6 and IL-8 was decreased sharply in IL-1β-treated SW982 cells (**Figures 4A,B**). These results demonstrated that FSGTC might promote the anti-inflammatory function in IL-1β-treated SW982 cells.

### Effects of FSGTC on the Inflammatory Expression of *IL-6* and *IL-8* Genes in IL-1β-Induced SW982 Cells

To examine the protective effect on the expression of inflammatory genes, the cells were treated with different doses (200 and 400 mg/mL) of FSGTC for 1 h before stimulation with IL-1β (10 ng/mL) for 6 h; then, the mRNA levels of *IL-6* and *IL-8* were measured by RT-qPCR. After stimulation with IL-1β, the mRNA expression levels of *IL-6* and *IL-8* were apparently increased. However, FSGTC decreased the level of expression of inflammatory genes, *IL-6* and *IL-8*, in IL-1β-stimulated SW982 cells (**Figures 4C,D**).

### Effects of FSGTC on IL-1β-Induced MAPK Signaling Pathways

**FIGURE 5:**
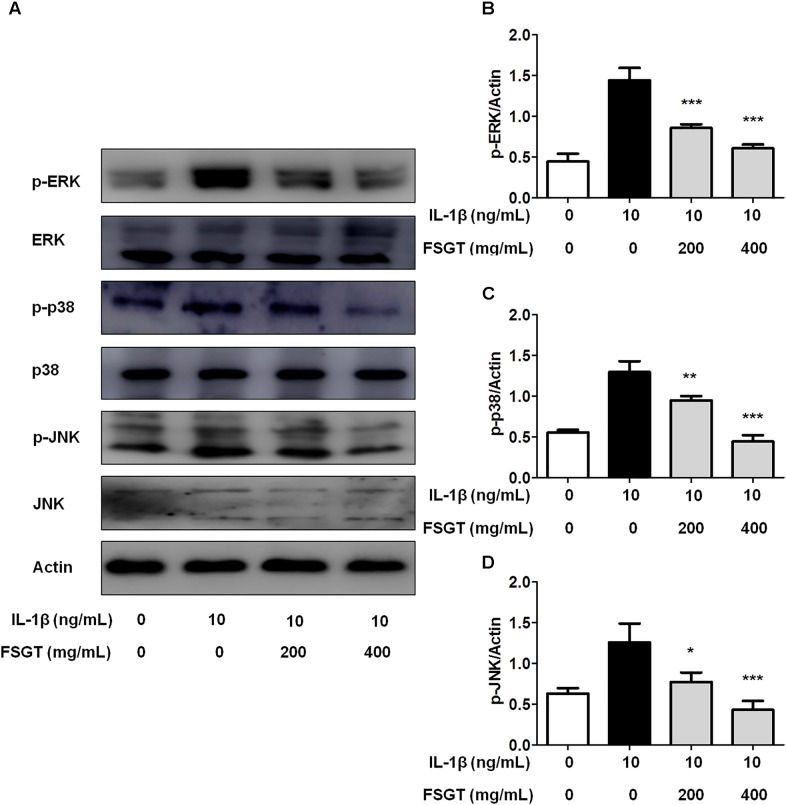
Effects of FSGTC on IL-1β-induced phosphorylation of ERK, p38, and JNK in SW982 cells. The cells were treated with various doses (200 and 400 mg/mL) of FSGTC. Lysates were prepared and Western blot performed using specific phospho-ERK, phospho-p38, and phospho-JNK antibodies **(A)**. Histogram analysis for the expression levels of p-ERK **(B)**, p-p38 **(C)**, and p-JNK **(D)** as described above. Actin was used as an internal control. *n* = 3, *x* ±*s*. ^∗^*p* < 0.05; ^∗∗^*p* < 0.01, ^∗∗∗^*p* < 0.001 vs. IL-1β without FSGTC.

The MAPK pathways, including ERK, JNK, and p38, play a key role in proliferation, apoptosis, and IL-1β-stimulated inflammation (Kaminska, 2005; Liu et al., 2015). To elucidate whether FSGTC inhibits MAPK activation, the phosphorylation of ERK, JNK, and p38 was examined by Western blot. We found that FSGTC treatment inhibited the phosphorylation levels of ERK, p38, and JNK (**Figures 5A–D**). As mentioned above, FSGTC could modulate the MAPK pathway for restricting the inflammatory response in IL-1β-treated SW982 cells.

### Effects of FSGTC on IL-1β-Induced NF-κB Signaling Pathway

**FIGURE 6:**
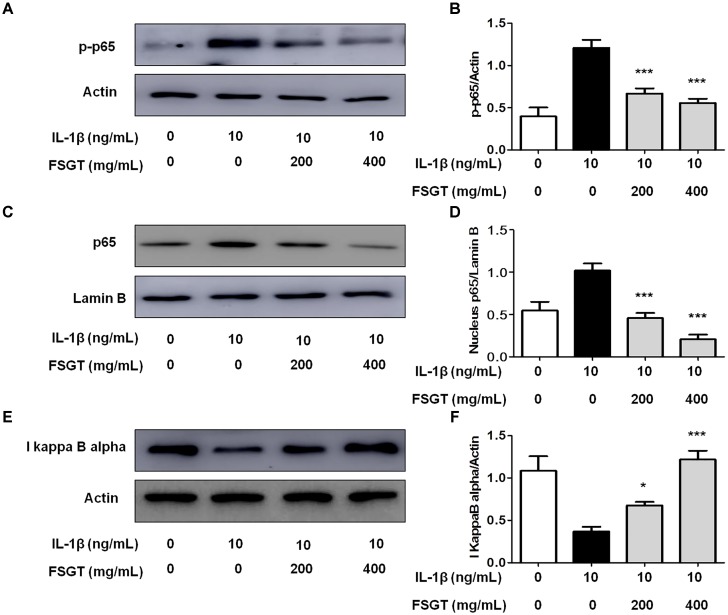
Effects of FSGTC on IL-1β-induced activation of NF-κB in SW982 cells. The cells were treated with various doses (200 and 400 mg/mL) of FSGTC. Lysates were prepared and Western blot performed using specific phospho-p65 antibodies **(A)**, NF-κB p65 **(C)**, and IκBα **(E)**. Histogram analysis for the expression levels of phospho-p65 **(B)**, nucleus p65 **(D)**, and IκBα **(F)** as described above. Actin and lamin B were used as internal controls. *n* = 3, *x* ± *s*. ^∗^*p* < 0.05; ^∗∗^*p* < 0.01, ^∗∗∗^*p* < 0.001 vs. IL-1β without FSGTC.

The members of the NF-κB transcription family can effectively modulate the transcription of numerous inflammatory mediators, and thus, is necessary for the production of cytokines and proteases by OA (Baier et al., 2003; Hah et al., 2010; Zhou et al., 2010). To investigate the anti-inflammatory mechanism of FSGTC, we examined the activation of NF-κB. FSGTC significantly reduced the IL-1β-induced degradation of IκBα (**Figures 6E,F**) and inhibited the phosphorylation (**Figures 6A,B**) and nucleus translocation of NF-κB p65 (**Figures 6C,D**). Taken together, we found that FSGTC regulated the NF-κB pathway to suppress the inflammatory response in IL-1β-treated SW982 cells.

### Effects of FSGTC on IL-1β-Induced AP-1 in SW982 Cells

**FIGURE 7:**
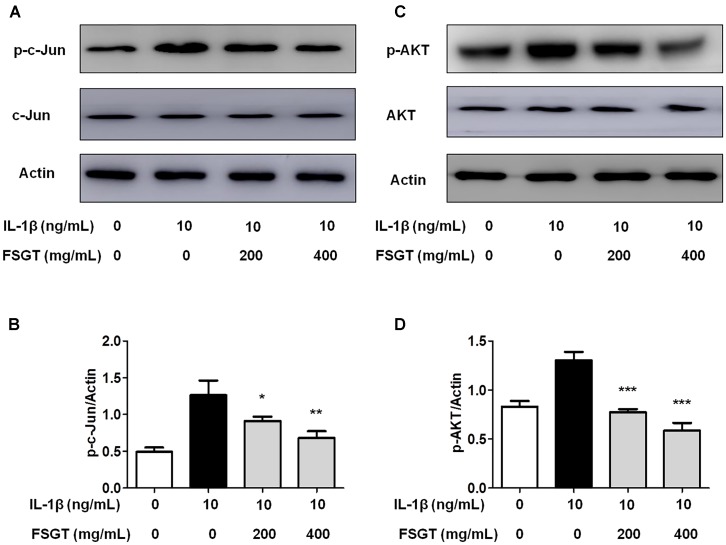
Effects of FSGTC on IL-1β-induced activation of AP-1 and Akt in SW982 cells. The cells were treated with various doses (200 and 400 mg/mL) of FSGTC. Lysates were prepared and Western blot performed using specific phospho-c-Jun **(A)** and phospho-Akt antibodies **(C)**. Histogram analysis for the expression levels of phospho-c-Jun **(B)** and phospho-Akt **(D)** as described above. Actin was used as an internal control. *n* = 3, *x* ±*s*. ^∗^*p* < 0.05; ^∗∗^*p* < 0.01, ^∗∗∗^*p* < 0.001 vs. IL-1β without FSGTC.

The function of transcription factor activator protein-1 (AP-1), as well as NF-κB, is to modulate the transcriptional regulation during inflammatory responses (Shiozawa and Tsumiyama, 2009). Therefore, we investigated the function of FSGTC on IL-1β-induced phosphorylation of c-Jun. The cells treated with FSGTC inhibited the phosphorylation level of c-Jun (**Figures 7A,B**). The data indicated that FSGTC modulated the AP-1 pathway to inhibit the inflammatory response in IL-1β-treated SW982 cells.

### Effects of FSGTC on IL-1β-Induced Akt Signaling Pathway

Previous reports showed that the control of inflammatory cytokines such as IL-6 is Akt signal-dependent (Malemud, 2015). We determined whether FSGTC effectuated the Akt activation. The results showed that the treatment with FSGTC inhibited IL-1β-induced phosphorylation of Akt in SW982 cells by regulating the Akt pathway (**Figures 7C,D**).

## Discussion

Osteoarthritis is a common chronic inflammatory disease caused by multiple molecular abnormalities (Bijlsma et al., 2011). Several clinical studies have shown that Chinese herbal monomers, such as pentacyclic triterpenoid, flavonoids, and single chalcone glycosides have efficient therapeutic effects in treating OA (Nguyen and Marquis, 2011; Shen et al., 2014; Cheng et al., 2016). Moreover, herbal remedies have the characteristics of multi-component and multi-target compounds such that they are prevalent and effective in the treatment of chronic illnesses in some Asian countries (Manheimer et al., 2009). Thus, investigating the mechanism of Chinese herbal remedies for the efficiency against OA is valuable.

The compounds from FSGTC included seven herbs, some of which can dispel the pathogenic cold and dehumidification, while others relieve pain. The overall effect is that the components complement and coordinate with each other to maximize the drug use (Chen et al., 2003; Liang et al., 2011). Therefore, it is necessary to elucidate the mechanism of the OA treatment with FSGTC. However, the beneficial effects of FSGTC on OA remain unclear. In the present study, we firstly demonstrated that FSGTC suppressed the production of inflammatory mediators *in vivo* and *in vitro*. It can also improve matrix degradation, as well as, chondrocytes and articular cartilage thickness *in vivo*. The phosphorylation of MAPK (ERK, p38, and JNK), c-Jun, and Akt and the nuclear translocation of NF-κB were suppressed by FSGTC *in vitro* (**Supplementary Figure S1**). These findings proposed that FSGTC can be used as a new therapeutic agent for the management of OA.

The release of IL-6, IL-8, and TNF-α plays a pivotal role in the synovial fluid and sera from patients with OA (Kaneko et al., 2000). The pro-inflammatory cytokine IL-6 is a major factor that results in the degradation of cartilage and bone, the influx of inflammatory cells, perpetuation of inflammation, and the emergence of rheumatoid factors (Szekanecz and Koch, 2016; Tanaka, 2016). The function of IL-8 is massive infiltration of immune cells and leukocyte recruitment. Also, microarchitecture resembles lymphoid tissue hypertrophic synovial tissue and angiogenesis (Guha and Mackman, 2001; Xagorari et al., 2002). TNF-α is primarily produced by activated macrophages and considered a vital pro-inflammatory cytokine in OA; it is also responsible for inflammation and joint destruction (Mateen et al., 2016). These results showed that FSGTC has potential anti-inflammatory influence and relieves cartilage damage in OA.

The MAPK family members, including ERK1/2, p38, and JNK, are the downstream targets of LPS-induced inflammatory cascades in macrophages. In the pro-inflammatory cytokine production process, the different MAPK signaling cascades regulate various steps (Kaminska, 2005). In this study, the results showed that MAPKs might be related to the suppression effects of FSGTC against the production of IL-6 and IL-8 in IL-1β-treated SW982 cells. The Akt signaling pathway is related to the release of inflammatory mediators and the destruction of joints in the pathogenesis of OA (Wu and Mohan, 2009). These results suggested that FSGTC may act as a therapeutic drug by inhibiting the cytoplasmic MAPK and Akt signaling pathways in OA treatment.

Nuclear factor kappa B, a major transcription factor, is involved in the production of cytokines and proteases (Baier et al., 2003; Hah et al., 2010). The present study demonstrated that FSGTC suppressed IκBα degradation and p65 protein translocation into the nucleus in IL-1β-treated SW982 cells. The AP-1 transcription factor is comprised of a homo- or heterodimeric protein complex that includes different Fos (c-Fos, Fra-1, Fra-2, and FosB) and Jun (c-Jun, JunB, and JunD) subfamilies (Hess et al., 2004). This study demonstrated that FSGTC inhibited the phosphorylation level of c-Jun in IL-1β-stimulated SW982 cells, thereby suggesting that FSGTC may act as a therapeutic drug by inhibiting the activation of NF-κB and AP-1.

## Conclusion

In the current study, we demonstrated the beneficial influence of FSGTC in an experimental OA model, which might be mediated through reduced expression of inflammatory cytokines via MAPK, NF-κB, AP-1, and Akt pathways. Both *in vitro* and *in vivo* studies confirmed the protective effects of FSGTC in the OA rat and cell models. These findings might demonstrate that FSGTC can be used as an effective therapeutic strategy for the alleviation of chronic inflammatory diseases in a clinical setting.

## Ethics Statement

This study was carried out in accordance with the Principle of Laboratory Animal Care (NIH Publication No. 85-23, revised 1985). The protocol was approved by the Animal Ethics Committee of Xinxiang Medical University.

## Author Contributions

B-FC designed the experiments. Y-XG carried out the experiments and wrote the manuscript. H-HY, CH, ML, D-DG, and J-JL helped in animal feeding and content detection experiment. B-FC, LW, H-JY, MW, and Z-WF designed, supervised, and corrected the manuscript.

## Conflict of Interest Statement

The authors declare that the research was conducted in the absence of any commercial or financial relationships that could be construed as a potential conflict of interest.

## Supplementary Material

FIGURE S1 Summary of anti-inflammatory mechanisms of FSGTC.
