# Supplementary figures and images for: Fengshi Gutong Capsule Attenuates Osteoarthritis by Inhibiting MAPK, NF-κB, AP-1, and Akt Pathways
Source: Front Pharmacol. 2018 Aug 17;9:910. doi: 10.3389/fphar.2018.00910 (PMC6107711; doi:10.3389/fphar.2018.00910)

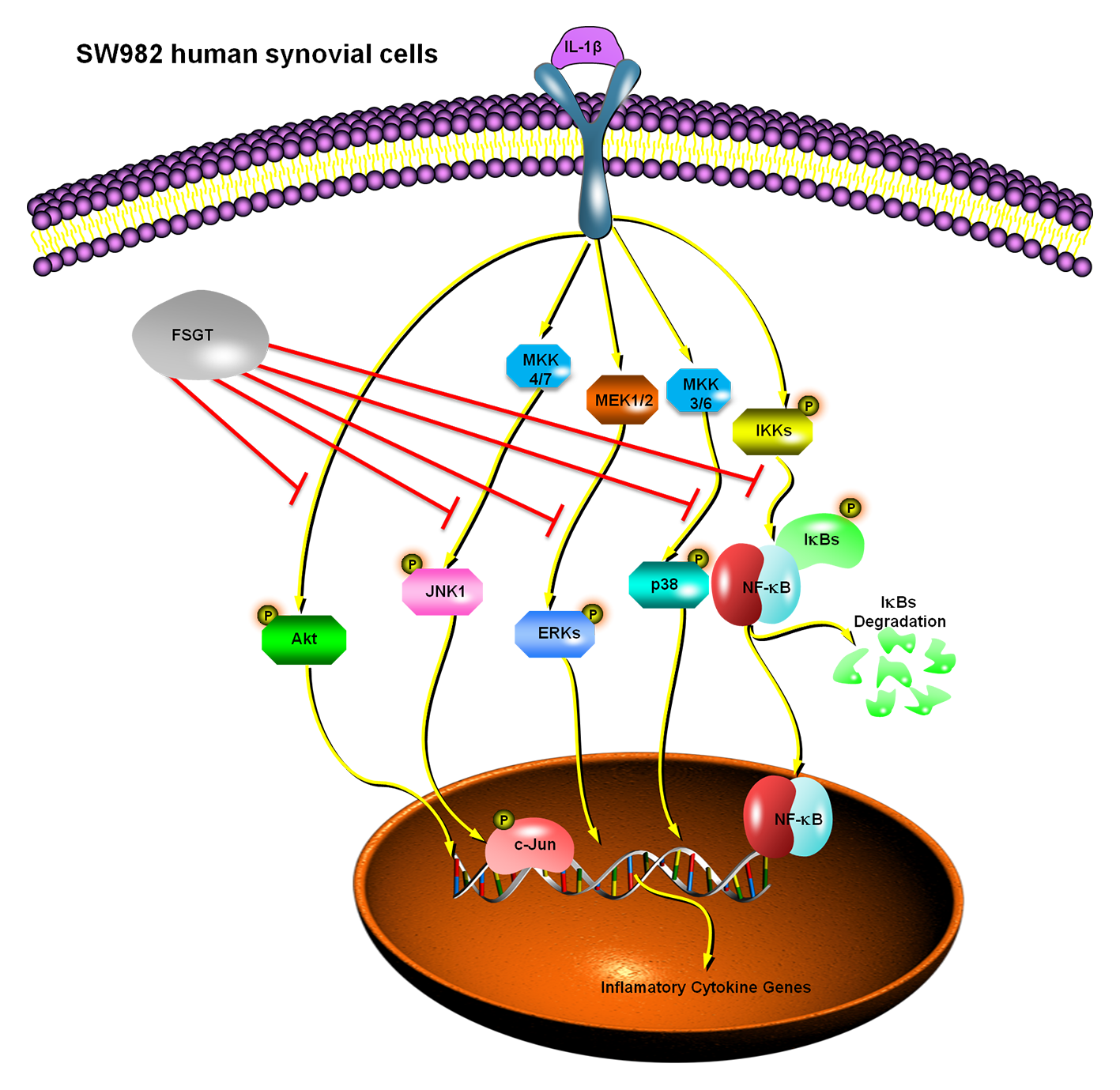

Supplement: FIGURE S1 [file Image_1.tif]
